# The Struggle for Life

**Published:** 1897-06

**Authors:** 


### The Struggle for Life.

The discoveries of Pasteur, Koch and others who have devoted themselves to the development of the science of bacteriology, have brought us face to face with the fact that man is constantly surrounded by multitudes of minute and potent enemies of life, and that he is only able to prolong his existence by the maintenance of a constant warfare. How this struggle for life is carried on has been very clearly shown by Metchnikoff and others who have penetrated deeply into the mysteries of cell life, and have actually been eye-witnesses to the combat between the living animal cells and the assailing microbes. To one who has been acquainted with the properties of microbes, with the terrific virulence which they sometimes possess, and the almost incredible potency of the poisons produced by them, it is indeed a marvel that any human beings or animals forms of any sort are able to exist. A deeper study of the subject, however, reveals to us the fact that, while man is thus surrounded by the most deadly and potent enemies of life, he is, nevertheless, by nature—so long as he conforms perfectly to the laws of health, thereby retaining a state of physical soundness—able successfully to resist the attacks of all known germs.—*Modern Medicine.*

